# Investigation of horizontal gene transfer of pathogenicity islands in *Escherichia coli *using next-generation sequencing

**DOI:** 10.1371/journal.pone.0179880

**Published:** 2017-07-21

**Authors:** Maxim Messerer, Wolfgang Fischer, Sören Schubert

**Affiliations:** Max von Pettenkofer-Institut für Hygiene und Medizinische Mikrobiologie, München, Germany; Animal and Plant Health Agency, UNITED KINGDOM

## Abstract

Horizontal gene transfer (HGT) contributes to the evolution of bacteria. All extraintestinal pathogenic *Escherichia coli* (ExPEC) harbour pathogenicity islands (PAIs), however relatively little is known about the acquisition of these PAIs. Due to these islands, ExPEC have properties to colonize and invade its hosts efficiently. Even though these PAIs are known to be acquired by HGT, only very few PAIs do carry mobilization and transfer genes required for the transmission by HGT. In this study, we apply for the first time next-generation sequencing (NGS) and *in silico* analyses in combination with *in vitro* experiments to decipher the mechanisms of PAI acquisition in ExPEC. For this, we investigated three neighbouring *E*. *coli* PAIs, namely the high-pathogenicity island (HPI), the *pks* and the *serU* island. As these PAIs contain no mobilization and transfer genes, they are immobile and dependent on transfer vehicles. By whole genome sequencing of the entire *E*. *coli* reference (ECOR) collection and by applying a phylogenetic approach we could unambiguously demonstrate that these PAIs are transmitted not only vertically, but also horizontally. Furthermore, we could prove *in silico* that distinct groups of PAIs were transferred "*en bloc*" in conjunction with the neighbouring chromosomal backbone. We traced this PAI transfer *in vitro* using an F' plasmid. Different lengths of transferred DNA were exactly detectable in the sequenced transconjugants indicating NGS as a powerful tool for determination of PAI transfer.

## Introduction

Evolution of bacteria occurs mainly in two major ways, vertical and horizontal. While the vertical transfer is rather slow and inconsistent, the horizontal transfer affects larger parts of the genome and has a greater influence on the evolution of bacteria, especially on the gain of pathogenic properties. Horizontal gene transfer (HGT) can take place by transduction, transformation and conjugation. Plasmids and also larger parts of the genome, like genomic islands, can be conjugated from one bacterium to another [1]. Pathogenicity islands (PAIs) are a subgroup of genomic islands. PAIs encode several virulence factors such as adhesins, toxins, capsules and siderophore systems and play a major role in the evolution of pathogenic bacteria such as extraintestinal pathogenic *Escherichia coli* (ExPEC). ExPECs are responsible for pyelonephritis, cystitis, septicaemia and newborn meningitis [2].

The species *E*. *coli* is subdivided into four major phylogenetic groups (A, B1, B2 and D). ExPECs belong mostly to groups B2 and D [2]. The *E*. *coli* reference (ECOR) collection consisting of 72 strains has been shown to represent the genetic diversity of this species. This collection of commensal and pathogenic strains from all phylogenetic groups of *E*. *coli* was composed in the early 1980s [3].

ExPECs harbour different PAIs, some of which are larger than 100 kb in size [4]. These islands have distinct structural features, e.g. they (i) are integrated at a tRNA gene, (ii) carry a gene for a phage-type integrase and (iii) display a GC-content distinct from that of the chromosomal backbone.

We focused on the high-pathogenicity island (HPI), the *pks* and the *serU* island. These PAIs contribute significantly to the ExPEC virulence [5–7], are next to each other on the chromosome and are not self-transmissible. Therefore, these PAIs are very suitable to investigate large scale HGT within the species *E*. *coli*.

The HPI is a widespread PAI among *Enterobacteriaceae* and has already been successfully used to demonstrate HGT [5]. This archetypal PAI encodes the synthesis of the siderophore yersiniabactin, representing a highly efficient iron-scavenging molecule. The sequence of the HPI is conserved among different bacterial species, with two distinct types of the island existing in ExPECs: approximately one percent of HPI-positive *E*. *coli* strains harbour an ICE-type (integrative conjugative element) island, which is completely self-transmissible. About 99% of *E*. *coli* strains carry a non self-transmissible island with a deletion of about 30 kb, encompassing the mobilization and transfer genes [5].

The two other PAIs, the *serU* island and the *pks* island, carry neither mobilization nor transfer genes [6;7]. The hybrid non-ribosomal peptide-polyketide colibactin encoded by the *pks* island induces double-strand DNA breaks and cell cycle arrest in eukaryotic cells [8]. The virulence factor TcpC encoded by the *serU* island interferes with the innate immune response by interrupting the NF-κB signalling pathway [9]. These two islands are only found in strains of the phylogenetic group B2.

For this study, we sequenced for the first time in large scale the whole genomes of the ECOR collection and some additional strains with next-generation sequencing (NGS). We used two approaches to investigate how HGT contributes to the evolution of PAIs. First, we examined the linked transfer of the described islands and its impact on evolution within the ECOR collection. With NGS, it was possible to compare the phylogeny of the whole PAIs and their neighbouring genomic regions in large scale. Second, we proved the co-transfer of these PAIs with an F' plasmid-mediated conjugation. It was possible to regard potential crossing-over regions for the F' plasmid, to see whether the DNA was conjugated in one or more pieces and to get an overview about the sizes of the transferred DNA.

## Materials and methods

### Bacterial strains, plasmid and primers

The entire 72 strains of the ECOR collection were used as a major set for the NGS approach and the subsequent *in silico* investigation of the phylogeny of *E*. *coli*. Further *E*. *coli* strains characterized previously were included in the sequencing project to complement the set of *E*. *coli* isolates: the strains S107 and S108 from the Le Gall collection [10] reveal a distinct *serU* island [7], the UPEC strain NU14 [11] was successfully used as donor strain in transfer experiments [5]. Finally, we included the archetypal UPEC strain 536 [12]—harboring all three analyzed PAIs—as a reference sequence for the phylogenetic analyses and as donor for the transfer experiments. The K-12 *E*. *coli* strain MG1655 (str^R^, phylogenetic group A, no β-hemolysis) [13] was used as recipient strain, as well as its nalidixic acid resistant mutant, which we constructed for this study. The F' plasmid (tet^R^) used in this study was isolated from laboratory *E*. *coli* strain XL1-Blue MRF' (Stratagene; Santa Clara, CA, USA). The primers used in this study are given in Table 1.

**Table 1:** Primers.

Primer name	Primer sequence
ChuA.1	5’-GACGAACCAACGGTCAGGAT-3’
ChuA.2	5’-TGCCGCCAGTACCAAAGACA-3’
YjaA.1	5’-TGAAGTGTCAGGAGACGCTG-3’
YjaA.2	5’-ATGGAGAATGCGTTCCTCAAC-3’
TspE4C2.1	5’-GAGTAATGTCGGGGCATTCA-3’
TspE4C2.2	5’-CGCGCCAACAAAGTATTACG-3’
fyuA.1080.for	5’-CTACGACATGCCGACAATGCC-3’
fyuA.1709.rev	5’-TGCTTCCCGCGCCATAACGTG-3’
clbA.IHE.for	5’-TAACTTCCTTCACTATCTCA-3’
clbA.IHE. rev	5’-GAGAGGCTAATGCGAGAAAT-3’
tcpC.for	5’-GGCAACAATATGTATAATATCCT-3’
tcpC.rev	5'-GCCCAGTCTATTTCTGCTAAAGA-3'
HPI-fyuA-2947.rev	5`-CAACTGCTTCCGTTATAGTGAC-3
HPI-fyuA-2132.for	5`-AAATTGCGATTAGGACAAATAG-3
p34S-Cm2.484.rev	5´-TCACCGTAACACGCCACATCTT-3´

The Primers which were used in this study are listed. They were used to determine the phylogenetic group (ChuA, YjaA, TspE4C2), to check the presence of the PAIs (*fyuA*, *clbA*, *tcpC*) and the insertion of a chloramphenicol resistance cassette (HPI-*fyuA*, p34S-Cm2).

### Whole genome sequencing and phylogenetic analysis

The genomic DNA was isolated using the "High Pure PCR Template Preparation Kit" (Roche Diagnostics; Unterhaching, Germany) as indicated by manufacturer’s protocol. The sequencing was performed at the Institute Pasteur, Paris (GENOPOLE—Transcriptomics & Epigenomics platform). To construct the libraries, the "TruSeq Kit" (Illumina; San Diego, CA, USA) was used according to manufacturer's instructions. The read type of the HiSeq 2000 (Illumina; San Diego, CA, USA) was single-end 100 nucleotides.

The parameters used for each approach with the NGS data are given in S2 Table.

The raw reads were imported as Illumina data to CLC Genomics Workbench 6.5 (CLC bio; Aarhus, Denmark). After trimming of the sequences, we performed *de novo* assemblies and alignments.

The phylogenetic trees (Maximum Likelihood (ML) with bootstrap and with Bayesian branch support) were constructed using the online tool PhyML 3.0 [14]. The CLC software was also used for the *in silico* MLST applying the Neighbour Joining (NJ) algorithm [15] and to create phylogenetic trees. The trees using ML with Bayesian inference and the analysis of the *E*. *coli* core genome are present in the manuscript. The trees using NJ and ML with bootstrap are attached to the supplemental section. The selected bootstrap cut-off is 75.

The statistical analysis was performed with the CLC software using the "Create Pairwise Comparison" tool. In order to calculate the DNA homology we used the parameter "percent identity" and to determine the number of Single Nucleotide Polymorphisms (SNPs) the parameter "differences". The software was also used to differentiate between donors and recipients DNA in the genomes of transconjugants of *in vitro* transfer experiments.

The draft genomes were annotated by the PGAP tool from NCBI. The *de novo* assemblies are deposited to NCBI GenBank and the NGS raw reads to NCBI SRA database. The accession numbers are listed in S1 Table.

Beside the PAIs and the neighbouring chromosome, housekeeping gene fragments and the *E*. *coli* core genome were investigated to definitively determine the phylogenetic groups. We used the Pasteur scheme which includes six housekeeping genes (*trpA*, *trpB*, *pabB*, *putP*, *icd* and *polB*) and has been used for several phylogenetic studies before [16;17].The core genome was analyzed using the tool Parsnp [18]. The closed genome of *E*. *coli* K-12 strain MG1655 was set as reference. The visualization of the core genome was performed by CLC Genomics Workbench.

### Conjugation and transfer of PAIs

For transfer experiments of the PAIs we used *E*. *coli* strains NU14 and 536 (phylogenetic group B2, β-hemolysis positive) as donors according to previous protocols [5]. As recipients, the *E*. *coli* strain MG1655 (phylogenetic group A, β-hemolysis negative) and its nalidixic acid (nal) resistance mutant were used. Dilutions were plated on LB plates containing chloramphenicol (cm) and streptomycin (str) or nalidixic acid to screen for transconjugants. Furthermore, the transconjugants were tested by PCR for the presence of the respective islands as well as the respective phylogenetic group. The β-hemolysis activity was checked on Columbia blood agar plates. The conjugation efficiency (colony forming units (cfu) per ml) was calculated as a ratio between the number of transconjugants and donors [19]. To calculate the efficiency for the transmission of the F' plasmid, we selected tetracycline- (tet) and str-resistant clones. All conjugations were done at least in triplicates for the estimation of efficiency.

$$conjugation\;efficiency=\frac{transconjugants}{donors}cfu/ml$$

## Results

### Simple analysis of draft genomes generated by large scale sequencing

The aim of this study was to decipher large scale horizontal gene transfer (HGT) in *Escherichia coli* affecting its genome. To analyse this, we determined for the first time the draft genomes of all the 72 strains of the *E*. *coli* reference (ECOR) collection as well as some additional isolates using next-generation sequencing (NGS). The raw data obtained by NGS consisted of a mean number of 9,105,077 reads per genome. A 99.84% of the sequenced nucleotides revealed unambiguous bases and the Phred quality score was 40 on average indicating high quality data [20]. Coverage of the genomes as well as N50 values are given in S1 Table. The *de novo* assembly using the CLC software resulted in about 150 contigs larger than 1 kb with maximum lengths of 145 to 430 kb. To prove the applicability of a phylogenetic approach using draft genomes, we deliberately resigned from performing any additional re-sequencing and gap closure procedures. In order to trace the horizontal transfer and evolution of PAIs, the focus of the present work was on the three neighbouring PAIs, namely the HPI, *pks* and *serU* island as well as the adjacent genomic regions [5;8;9].

With the generated NGS data we performed three approaches. Firstly, the investigation using an *in silico* Multi Locus Sequence Typing (MLST) based on different fragments of housekeeping genes in comparison with the *E*. *coli* core genome. Secondly, the analysis of the three mentioned neighbouring PAIs and their transmission *in silico*. Thirdly, the transfer of these three PAIs *in vitro* followed by an *in silico* study of the resulting transconjugants.

### Investigation of an MLST approach and the core genome to confirm the phylogeny of the *E*. *coli* species

In order to confirm the ECOR phylogeny and to distinguish between vertical and horizontal PAI transfer in *E*. *coli* we applied an *in silico* MLST approach and analyzed the species core genome. Several MLST schemes have been described so far to delineate the *E*. *coli* phylogeny including the Achtman and the Pasteur scheme [21;22]. These schemes rely on the sequence variation of distinct fragments of *E*. *coli* housekeeping genes with sequence length of about 500 bp to distinguish different phylogenetic groups. The NGS data enabled the comparison of the nucleotide sequences of the gene fragments of the known Pasteur MLST scheme and the *E*. *coli* core genome to classify the strains. As we investigated the possibility to work with draft genomes without re-sequencing, the tool Parsnp was suitable to analyse the *E*. *coli* core genome using incomplete genomes.

**Fig 1:**
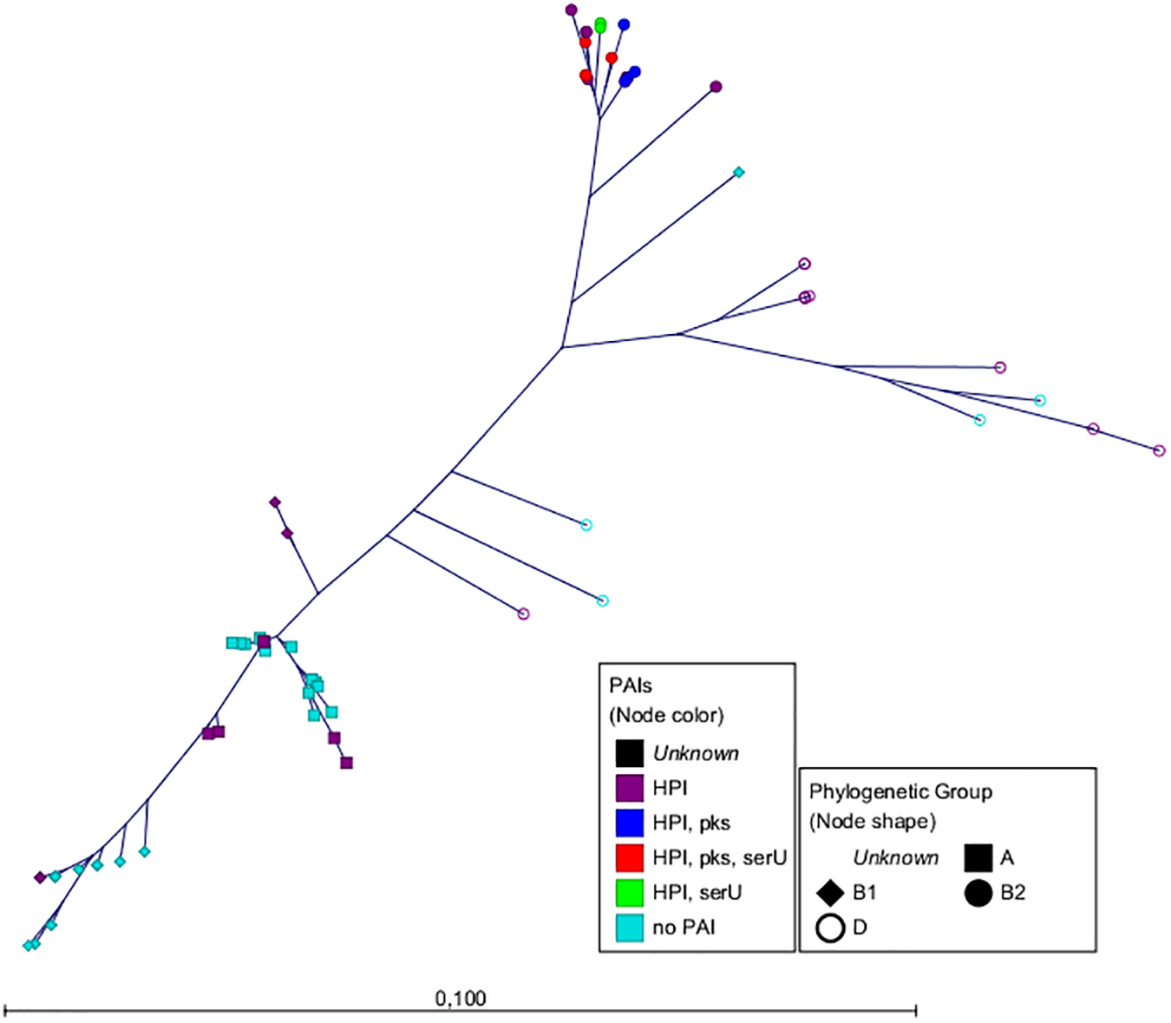
Radial tree of the six housekeeping gene fragments. The radial tree of the six housekeeping gene fragments (*trpA*, *trpB*, *pabB*, *putP*, *icd* and *polB*) from the ECOR collection and strains S107, S108 and 536 performed by PhyML using the Maximum Likelihood algorithm with Bayesian branch support. The scale bar represents the number of SNPs per nucleotide. The node colour represents the distribution of the PAIs. The node shapes show the phylogenetic group according to the triplex PCR [2].

The fragments of these six housekeeping genes from the Pasteur scheme led to concatenated sequences with a total length of 3,045 bp [16]. To generate an MLST-based tree, we compared the concatenated sequences from all strains using Maximum Likelihood (ML) [14] and the Neighbour-Joining (NJ) algorithm [15]. The constructed phylogenetic tree (Figs 1 and S1) termed "MLST tree" matched highly with previously published data [22–24].

**Fig 2:**
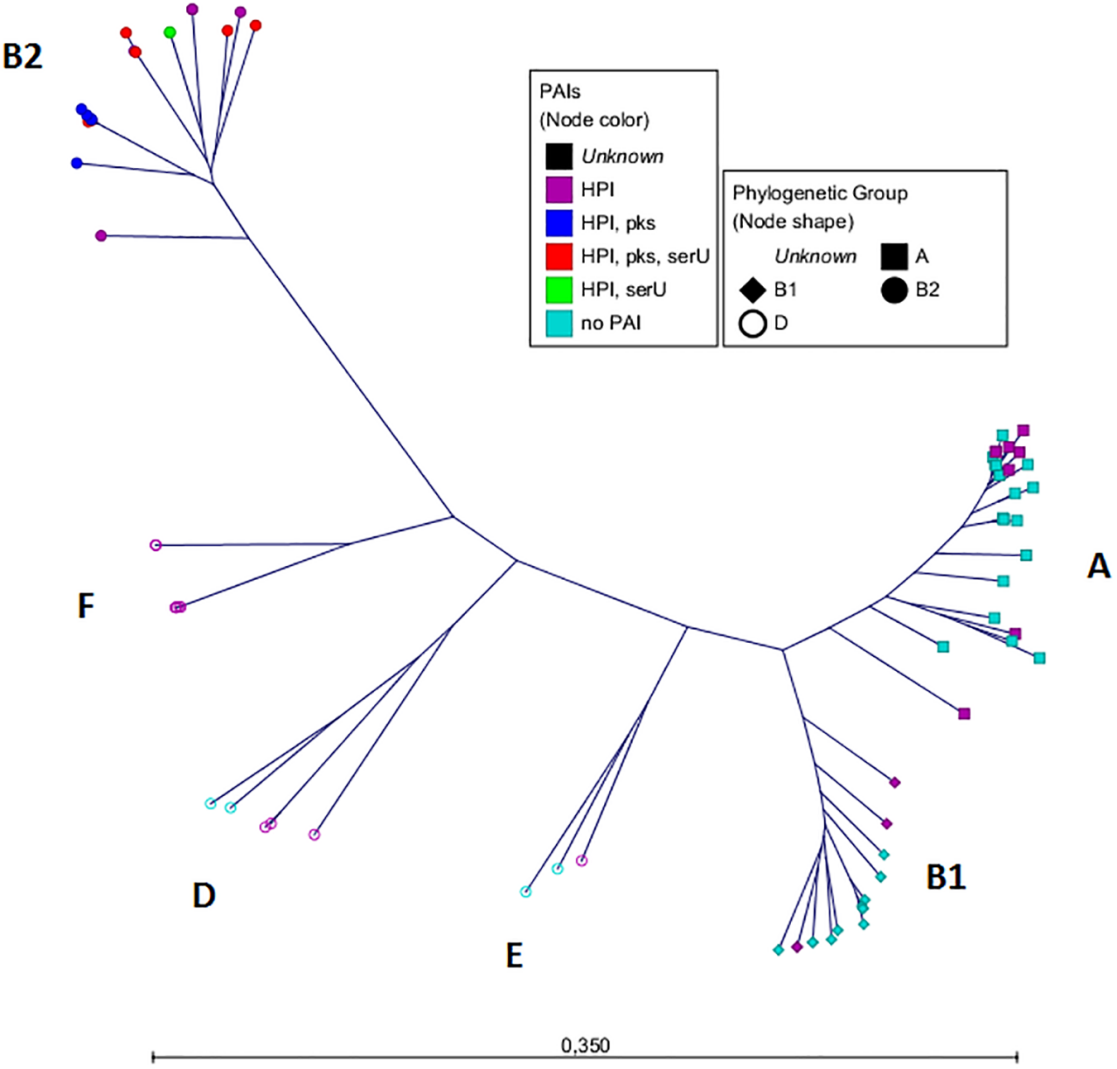
Radial tree of the *E*. *coli* core genome. The radial tree of the core genome was generated by Parsnp. Strain MG1655 was set as reference. The total coverage among all sequences was 40.9%. The phylogenetic groups are highlighted. The scale bar represents the number of SNPs per nucleotide. The node colour represents the distribution of the PAIs. The node shapes show the phylogenetic group according to the triplex PCR [2].

Next, we analyzed the *E*. *coli* core genome using the tool Parsnp [18] (Fig 2). As reference we set the closed genome of *E*. *coli* K12-strain (phylogenetic group A). The total coverage among all sequences representing the core genome was 40.9%. This is in total agreement with published data [25;26]. The phylogenetic groups revealed by the analysis of the core genome reflected mostly the groups shown by the "MLST tree". The strains were assigned to the mayor groups A, B1, D and B2 and also to the minor groups E and F [21;22], which are highlighted. The ECOR strain phylogeny is summarized in S3 Table.

### Specific subtypes of the different PAIs are correlated to specific groups

After the investigation of the *E*. *coli* phylogeny we examined the HGT of immobile PAIs *in silico*. The NGS data enabled a large scale analysis of the HPI, *pks* and *serU* island and their neighbouring chromosomal regions. The genome region under investigation covering all islands and backbone sequences in between encompassed about 126 kb. The draft genome sequences provided sufficient sequence information to analyse this DNA region in all strains.

**Fig 3:**
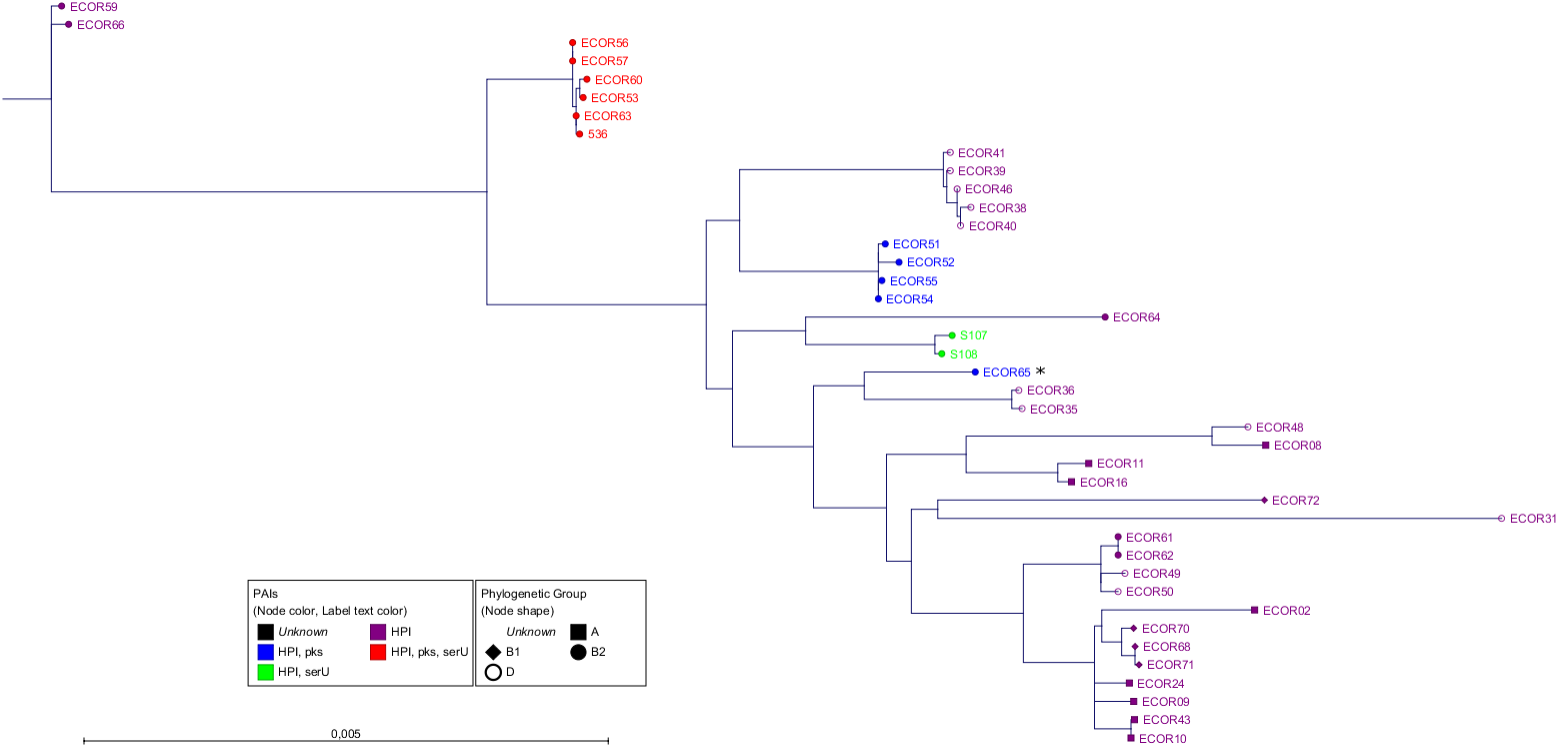
The phylogenetic tree of the entire HPI. All strains are at least HPI-positive. The text and dot colour represents the PAI-group and the dot shape the phylogenetic group. Except strain ECOR65 (asterisk) from PAI-group 2a, all members of PAI-groups 2a (blue), 2b (green) and 3 (red) showed a HPI subtype specific for their group. The utilized algorithm was Maximum Likelihood with Bayesian branch support performed by PhyML. The scale bar represents the percentage of SNPs per nucleotide. The length of the HPI sequence is about 31.5 kb. The average homology and SNPs within the PAI-groups: 2a 99.99% (4.5), 2b 99.98% (7), 3 99.99% (2.4). The average homology and SNPs between the PAI-groups: 2b-2a 99.63% (116.8), 2b-3 99.54% (144.2), 2a-3 99.59% (129.9). The average homology and SNPs between ECOR65 and the PAI-groups: EC65-2a 99.66% (107.3), EC65-2b 99.67% (104.5), EC65-3 99.53% (149.3).

First, we constructed three phylogenetic trees (NJ, ML with bootstrap and Bayesian inference) comparing the entire HPI (31.5 kb) of all positive strains in order to determine the phylogenetic history of this island (Figs 3 and S2). Interestingly, in all trees we could observe distinct clonal groups of the HPI related to the number and distribution of the neighbouring PAIs. Clonal groups encompassing strains with a distinct number of PAIs were named "PAI-groups". Strains of PAI-group 1 carried only the HPI. PAI-group 2a strains included the HPI and the *pks* island, PAI-group 2b strains harboured the HPI and the *serU* island. In members of PAI-group 3, all three islands were present. Notably, looking at the phylogram of the HPI (Figs 3 and S2), the formation of PAI-groups 2a and 2b was apparently not due to the deletion of single PAIs from PAI-group 3, as the members of the three different PAI-groups did not share the same clonal group of the HPI. Members of PAI-group 1 revealed different distinct HPI clonal groups as shown in the phylogenetic tree (Fig 3). This heterogeneity suggested that the HPI is the eldest of the three PAIs. Analyses for sequence homology of the entire HPI further corroborated the existence of these clonal groups. The analyses revealed that within the PAI-groups 2a, 2b and 3, the average homology was very high with values between 99.98% and 99.99% (4.5, 7 and 2.4 SNPs, respectively) (Fig 3). In contrast, the homology between different PAI-groups was considerably lower with sequence identities between 99.54% and 99.63% (116.8 to 144.2 SNPs). This indicated that these clonal groups of the HPI had a different phylogenetic history.

**Fig 4:**
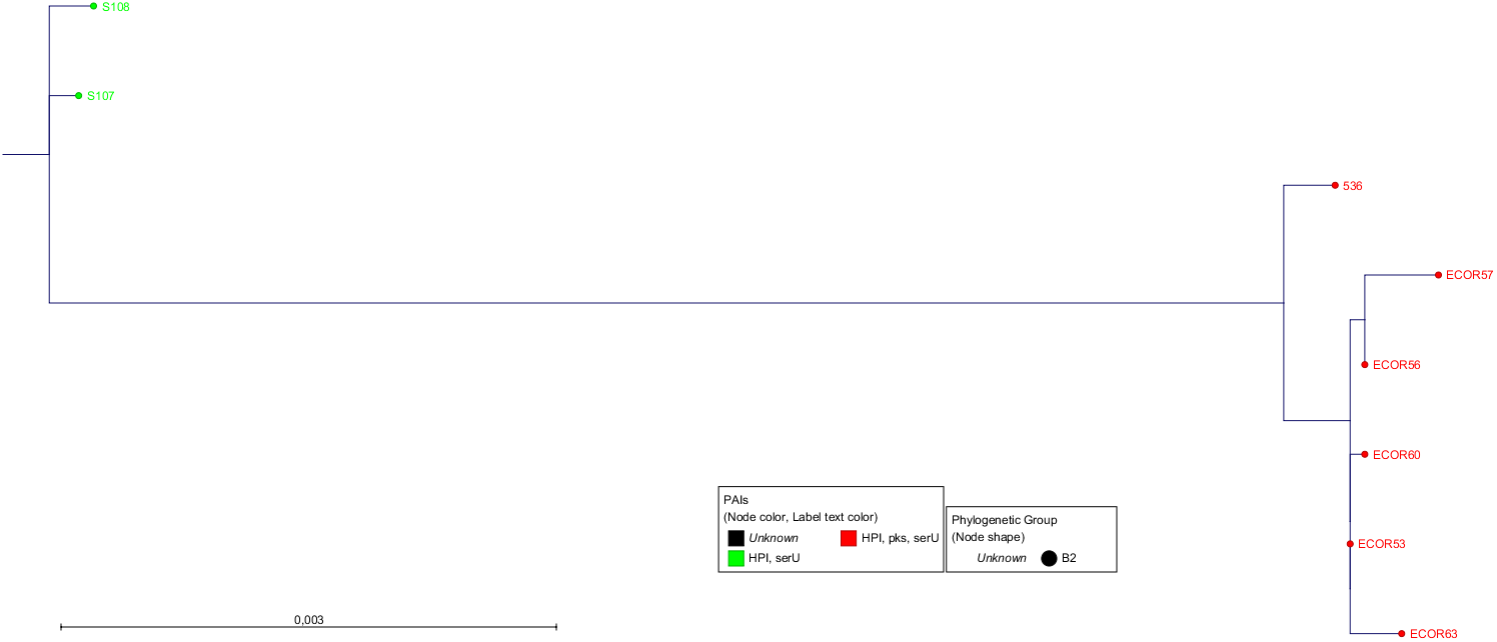
The phylogenetic tree of the entire *serU* island. All strains are at least HPI- and *serU* island-positive. The text and dot colour represents the PAI-group and the dot shape the phylogenetic group. The members of PAI-groups 2b (green) and 3 (red) showed a *serU* island subtype specific for their group. The algorithm which was used by PhyML was Maximum Likelihood with Bayesian branch support. The scale bar represents the percentage of SNPs per nucleotide. The size of the *serU* island is about 27 kb. The average homology and number of SNPs were similar within the PAI-groups for 2b and 3 with 99.93% (18.0) and 99.94% (16.6), respectively. Between these two PAI-groups, the homology was 99.27% with 197.3 SNPs on average.

**Fig 5:**
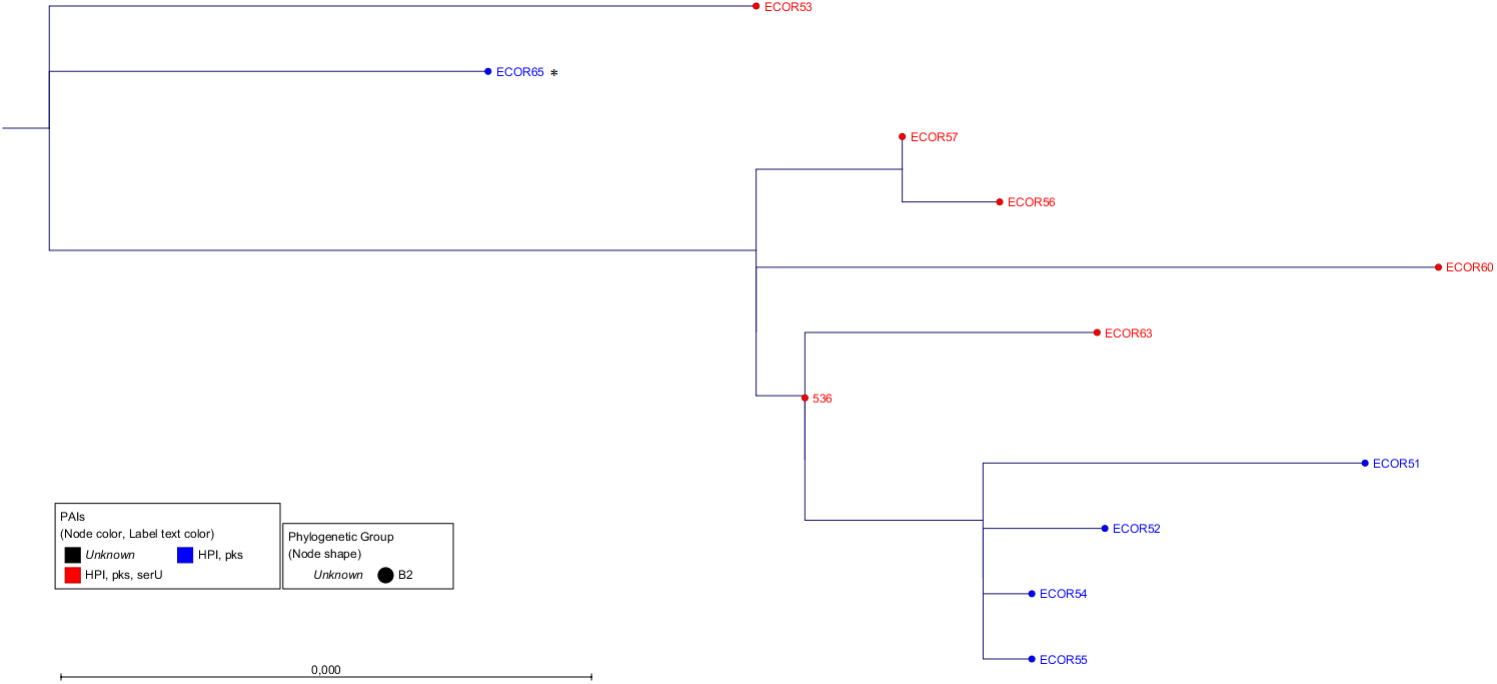
The phylogenetic tree of the entire *pks* island. All strains are at least HPI- and *pks* island-positive. The text and dot colour represents the PAI-group and the dot shape the phylogenetic group. Except strain ECOR65 (asterisk) from PAI-group 2a, all members of PAI-groups 2a (blue) and 3 (red) showed a *pks* island subtype specific for their group. The algorithm we used was Maximum Likelihood with Bayesian branch support performed by PhyML. The scale bar represents the number of SNPs per nucleotide. The sequence length of the *pks* island is about 54.5 kb. Within PAI-group 2a the homology and the number of SNPS on average are 99.99% and 5.7 respectively, within PAI-group 3 99.97% and 18.3. The average homology and SNPs between ECOR65 and the PAI-groups: EC65-2a 99.89% (61.8), EC65-3 99.89% (59.3).

Next, the *serU* island (27 kb) and *pks* island (54.5 kb) were each investigated regarding their relationship. The analysis of their genome sequences revealed that the respective *serU* islands (Figs 4 and S3) and *pks* islands (Figs 5 and S4) differed significantly according to the affiliation to the distinct PAI-groups. Each PAI-group consisted of a specific clonal group regarding the respective island. The sequence homology of *serU* islands of strains within PAI-group 2b was 99.93% (18 SNPs). A similar homology was found within PAI-group 3 with 99.94% (16.6 SNPs). However, comparing these two clonal groups, the lower homology of 99.27% (197.3 SNPs) corroborated a different evolution. Regarding the nucleotide sequences of the *pks* islands within PAI-groups 2a, the homology was 99.99% (5.7 SNPs). Within PAI-group 3, a sequence identity of 99.97% (18.3 SNPs) was found. Between these two PAI-groups, the homology was also 99.97% (16 SNPs). Comparing the two different islands, the *pks* islands were in general more similar than the *serU* islands (Figs 4 and 5). This indicated that the *pks* island is probably the most recently acquired of the investigated PAIs.

**Fig 6:**
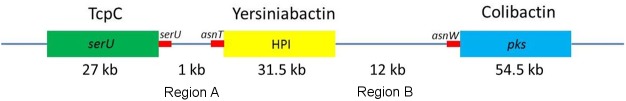
The arrangement of the PAIs on the chromosome. Each island is inserted in a tRNA (*serU* island: *serU* tRNA; HPI: *asnT* tRNA; *pks* island: *asnW* tRNA). The size is given in kilobases (kb). The regions between the PAIs are called inter-PAI regions (between the *serU* island and the HPI (region A): about 1 kb; between the HPI and the *pks* island (region B): about 12 kb).

**Fig 7:**
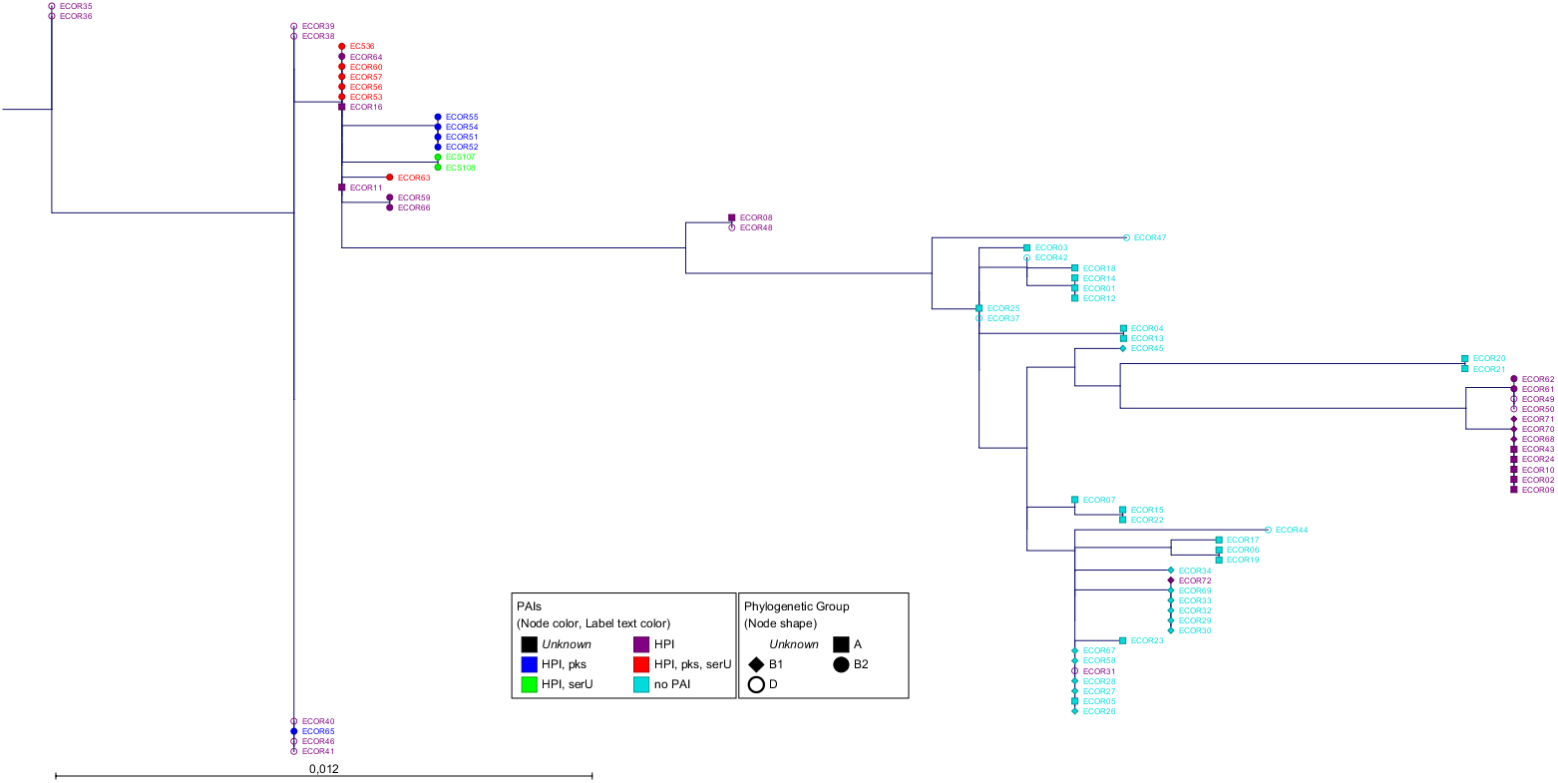
Phylogenetic tree of region A. The inter-PAI region between the *serU* island and the HPI (region A) is about 1 kb and is shown as phylogenetic tree. The text and dot colour represents the PAI-group and the dot shape the phylogenetic group. The algorithm which was used by PhyML was Maximum Likelihood with Bayesian branch support. The scale bar represents the percentage of SNPs per nucleotide. Within PAI-groups 2a and 2b, the percental homology is 100% without any SNP. Within PAI-group 3, the homology is 99.97% and the number of SNPs 0.3 on average.

**Fig 8:**
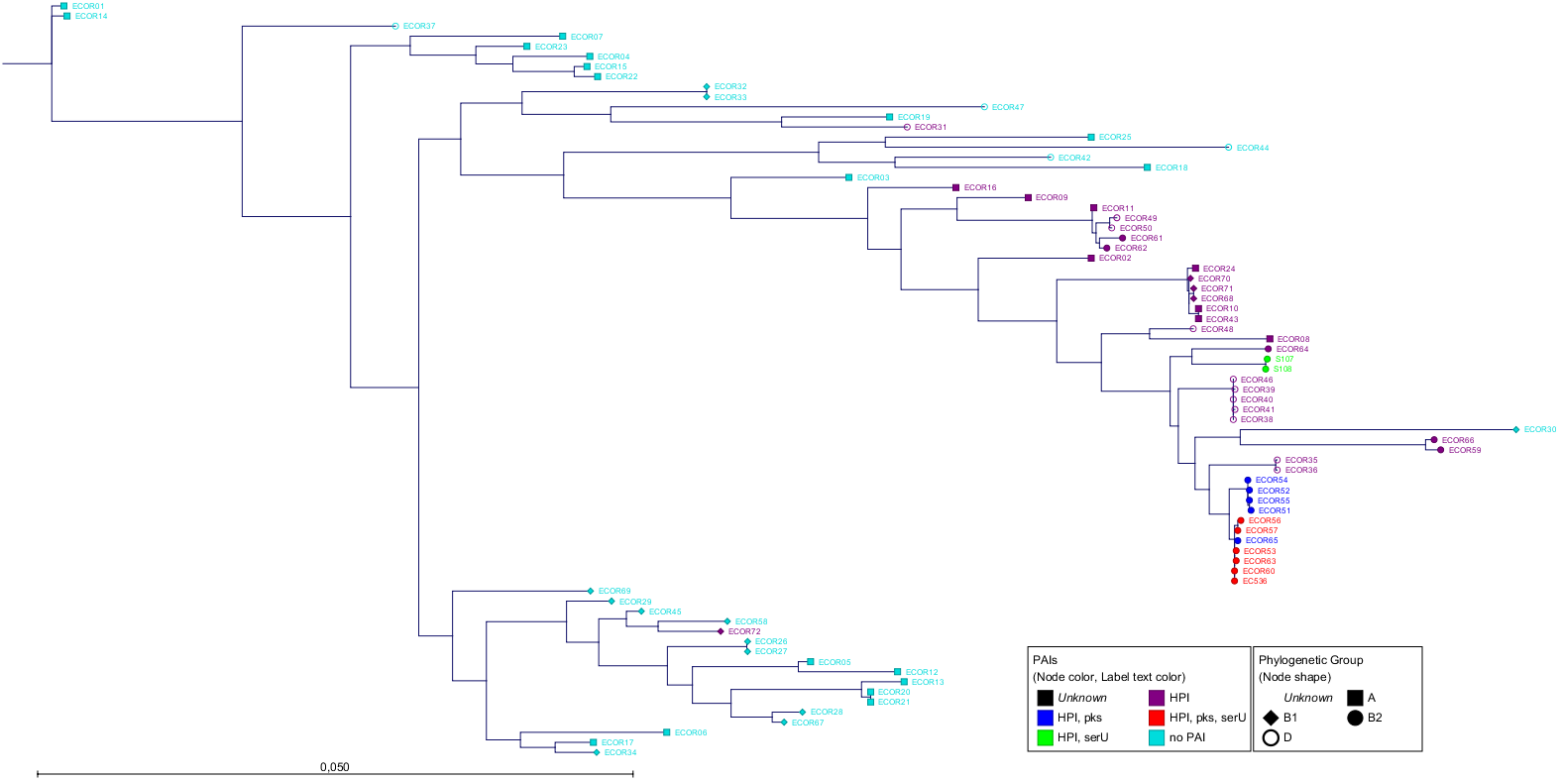
Phylogenetic tree of region B. The dendrogram of the inter-PAI region between the HPI and the *pks* island (region B). The size of the sequence is about 12 kb. The text and dot colour represents the PAI-group and the dot shape the phylogenetic group. The algorithm which was used by PhyML was Maximum Likelihood with Bayesian branch support. The scale bar represents the number of SNPs per nucleotide. For the PAI groups 2a, 2b and 3, the average homology was 99.99%,99.98% and 99.98% respectively and the number of SNPs were 1.3, 3 and 2.3 on average.

Moreover, we investigated the *E*. *coli* backbone genome between the islands to gain insight into the diversity of these sequences. If the PAIs were transferred together "*en bloc*", the backbone should cluster and congregate like the PAI subtypes. We named these sequences "inter-PAI regions" which exist between the *serU* island and the HPI (region A, 1 kb) and between the HPI and the *pks* island (region B, 12.5 kb) (Fig 6). We constructed phylogenetic trees (NJ, ML with bootstrap and Bayesian inference) out of these sequences which are shown in Figs 7 and S5 and Figs 8 and S6. These trees resembled that of the HPI-based tree identifying the same PAI-group and indicated that the respective backbone regions were transmitted together "*en bloc*" with the islands. The analyses of sequence homologies corroborated this hypothesis (Figs 7 and 8). The sequences of region A were almost identical within the PAI-groups 2a, 2b and 3 with sequence identities from 99.97% to 100%, whereas those between the PAI-groups were definitely lower (99.60% to 99.78%). Additionally, the sequences of region B within respective PAI-groups exhibited very high homologies (99.98% to 99.99%). These sequence homologies were less pronounced between the three PAI-groups (99.28% to 99.87%).

As third level of evidence, we wanted to ensure that the PAIs were transferred via HGT and not vertically through cell division. For this purpose we compared the phylogenetic tree of the core genome (Fig 2) with that of the PAIs (Figs 3–5). If the sequences of the core genome cluster together, the strains originated from the same ancestor. In contrast, a sequence variety indicates a different origin and supports the idea of a transmission of the PAIs *via* HGT. The fact that strains with the same PAI distribution did not seem to be clonal regarding their core genome sequences (Fig 2) proved that the transmission of the PAIs was not vertical, but horizontal.

### Analysis of the outlier strain ECOR65 which showed a distinct phylogenetic pattern

By analysing the ECOR collection *in silico*, we found one strain, namely ECOR65, which did not fit into the proposed scheme. ECOR65 harboured the PAIs HPI and *pks* island and was thus, by definition, a member of PAI-group 2a. Comparing the HPI sequence of ECOR65 with those of the PAI-groups 2a, 2b and 3, the HPI of ECOR65 was only distantly related (Fig 3). In contrast, looking at the phylogenetic trees of the *pks* island (Fig 5) and the inter-PAI regions (Figs 7 and 8), the strain resembled members of PAI-group 3, suggesting the loss of the *serU* island in ECOR65. To prove whether ECOR65 lost this island we divided the inter-PAI regions into equal parts and analysed them separately to examine the distribution of the genetic differences. The region between the *serU* island and the HPI (region A) is 1 kb in length. By investigating the two 500 bp parts of region A we found the part next to the HPI to cluster ECOR65 together with strains of PAI-group 3. In the other 500 bp part the sequence of ECOR65 had an aberration of only one single SNP compared to the sequences of the other strains, which we regarded as non-discriminatory. The length of the inter-PAI region between the HPI and the *pks* island (region B) is about 12.5 kb. We divided this region into equal sized parts and analysed the respective phylogenetic trees. The dendrogram of the 6 kb region next to the HPI classified ECOR65 strain to be of PAI-group 3. In contrast, the tree of the region next to the *pks* island clustered strains of PAI-group 3 and 2a together with ECOR65. However, the latter 6 kb region was regarded as non-discriminatory displaying only an average of 1.4 SNPs. In conclusion, these data pointed towards the loss of the *serU* island in the ECOR65 strain, but did not explain the sequence difference of HPI of ECOR65 to members of the PAI-group 3.

### PAIs are transmissible *via* F' plasmid-mediated transfer

After the *in silico* investigation of the ECOR collection and distinct additional strains, the hypothesis of an "*en bloc*" transfer was proven applying an *in vitro* approach. We performed an F' plasmid-mediated conjugation to reconstruct the simultaneous transmission of multiple PAIs. The resulting transconjugants were sequenced and further analyzed. We focused (i) on the amount of transferred DNA from different donor strains and (ii) on the recognition of potential hotspots for recombination. NGS is a powerful tool to retrace an "*en bloc*" transfer and to gain insight into the evolution of the PAIs and the surrounding backbone. The sequenced genomes of donors, transconjugants and recipients were compared by an alignment to identify the exact regions of homologous recombination.

We used both *E*. *coli* strain NU14 HPI-Cm F' carrying the HPI together with the *pks* island (PAI-group 2a) and *E*. *coli* 536 HPI-Cm F' harbouring all three islands (PAI-group 3) as donor strains to confirm our hypothesis. The respective HPIs were tagged with a chloramphenicol resistance cassette applying the method of Datsenko and Wanner [27] to track the transfer of the PAIs. As the investigated islands were immobile, an F' plasmid was conjugated to the donors to enable the transfer. As the transmissions were conducted to the recipient *E*. *coli* MG1655, the further characteristics of selected transconjugants were to belong to phylogenetic group A and to reveal no β-hemolysis on blood agar plates. In order to compare the conjugation efficiency of an F' plasmid transfer in general (str^R^, tet^R^) with a PAI transmission in special (str^R^, cm^R^), we calculated the ratio between the number of transconjugants and donors [19]. The transconjugants were checked by PCR for the presence of the HPI and the *pks* island, and in case of the donor strain 536 HPI-Cm F', additionally for the presence of the *serU* island.

The pure F' plasmid transfer exhibited a conjugation efficiency of 5.24 x 10^−4^ cfu/ml. None of 120 screened clones were resistant to chloramphenicol indicating a transmission of other DNA content than the HPI. In contrast to this conjugation, the HPI transfer rate of donor NU14 HPI-Cm F' (HPI and *pks* island) was significantly lower with 2.94 x 10^−7^ cfu/ml. The conjugation efficiency for the HPI transfer of donor 536 HPI-Cm F' (HPI, *pks* and *serU* island) was very similar with 3.85 x 10^−7^ cfu/ml. This indicated that the efficiency was independent of the donor. Interestingly, 50% of the transconjugants were tetracycline-resistant, indicating the retention of the F' plasmid. After three passages without antibiotic pressure 90% of the initially tetracycline-resistant strains were not able to grow on tetracycline-LB-plates any more. The loss of the resistance could reflect the recombination *via* double crossing-over (exchange of donor and recipient DNA) and the loss of the F' plasmid. Notably, the tetracycline-resistant transconjugants were able to spread their new PAIs with a 100-fold enhanced conjugation efficiency of 4.14 x 10^−5^ cfu/ml. This transfer rate resembled more the pure F' plasmid transfer than the PAI transfer.

Next, we analysed the transconjugants *in silico*. For this, we sequenced the whole genomes of the respective transconjugants to gain insight into the F' plasmid transfer of the PAIs, their backbone and the recombination into the recipients. We took tetracycline-sensitive transconjugants from five independent conjugations of donor NU14 HPI-Cm F' (PAI-group 2a) and from four independent conjugations of donor 536 HPI-Cm F' (PAI-group 3). The sequences of all investigated transconjugants revealed exclusively unfragmented DNA transfer events, with only one piece of foreign DNA found per isolate. Furthermore, the tetracycline-negative strains revealed no F' plasmid DNA in their genome indicating a double, rather than a single, crossing-over. The PAIs were always transmitted completely from donors to recipients. This could be due to the fact that no homologous regions (related to the islands) were present in the recipient. Also no IS elements contributing to recombination are described in the three PAIs.

With the entire genome sequences of donor and recipient strains in our hands, the NGS approach enabled us to distinguish between DNA of donor and recipient within the transconjugant sequences. The comparison of donors, recipients and transconjugants showed that the size of integrated DNA was highly variable (Table 2). The transferred DNA from the PAI-group 2a strain NU14 HPI-Cm F' varied between 131,132 bp and 421,058 bp. The PAI-group 3 strain 536 HPI-Cm F' transferred DNA fragments from 62,496 bp to 470,591 bp in size. This indicated that the size of integrated DNA was independent of the donor. Mostly, no regular hotspots for recombination of the F' plasmid within the chromosome were detectable. Although one integration site was similar in three transconjugants, the recombination took place at various locations within the genome. All sites were analysed for IS elements in the genome of *E*. *coli* MG1655, but none were found at these places. This finding could relate to the fact that recombination of an F' plasmid into the chromosome can occur at any homologous region [28].

**Table 2:** Transconjugants.

Transconjugant	size of donor DNA [bp]
NU14 HPI-Cm F’ x MG1655 K1	223,368–224,149
NU14 HPI-Cm F’ x MG1655 K2	321,955–322,073
NU14 HPI-Cm F’ x MG1655 K3	419,923–421,058
NU14 HPI-Cm F’ x MG1655 K4	131,132–132,269
NU14 HPI-Cm F’ x MG1655 K5 (no *pks* island)	198,334–198,400
536 HPI-Cm F’ x MG1655 K1	225,011–226,036
536 HPI-Cm F’ x MG1655 K2	470,189–470,591
536 HPI-Cm F’ x MG1655 K3	348,167–348,408
536 HPI-Cm F’ x MG1655 K4 (no *pks* island)	62,496–62,711

The transconjugants from the independent conjugations NU14 HPI-Cm F' x MG1655 and 536 HPI-Cm F' x MG1655 are listed. The amount of transferred donor DNA is given in base pairs (bp).

Interestingly, only 64 of 70 transconjugants (91.4%) analysed by PCR had obtained the *pks* island from the donors. No residual sequences of the *pks* island were found *in silico* in the two sequenced isolates which had previously been identified as PCR-negative. This indicated a transfer in an "all or nothing" fashion.

## Discussion

The 72 strains of the *Escherichia coli* reference (ECOR) collection were composed in the early 1980s from a selection of 2,600 *E*. *coli* isolates to represent the range of genotypic variation in the species as a whole [3]. Although the ECOR collection does not fully represent the different pathotypes of *E*. *coli*, it is suitable to analyse the horizontal gene transfer (HGT) of extraintestinal pathogenic *E*. *coli* (ExPEC) pathogenicity islands (PAIs) due to the presence of this pathotype in the compilation [24]. This strain collection was used for a variety of publications over the last three decades. Differences in some strains have been described within the ECOR collection, mainly regarding their published virulence factors [29]. Therefore, next-generation sequencing (NGS) is a powerful tool enabling the definite verification of the respective ECOR strains and delivering the adequate sequence data for phylogenetic comparisons of the strains. Meanwhile, the cost for whole genome sequencing decreased enormously compared to the introduction of this new technology [30]. Besides the ECOR collection, we used three archetypal ExPEC strains for our study: strain 536 [12], S107 and S108 [10]. As ExPECs are relevant clinical pathogens with virulence often linked to PAIs [31], they are ideal to investigate PAI transfer in *E*. *coli*.

To our knowledge, the HGT of large DNA regions has not been studied by an NGS approach in such a comprehensive strain collection. Due to the high quality raw data obtained by NGS, the assembly of draft genomes containing large contigs was possible. The aim of this study was to analyse if these NGS draft genomes are sufficient for (i) phylogenetic analyses of the core genome and (ii) defining PAI transfer in *E*. *coli* by combining *in silico* and *in vitro* experiments. In order to determine the phylogenetic groups of the ECOR collection and the additional strains, we analyzed the *E*. *coli* core genome and additionally applied an *in silico* MLST by analysing the housekeeping genes of the Pasteur MLST scheme (*trpA*, *trpB*, *pabB*, *putP*, *icd* and *polB*), which seemed to be only little affected by HGT and recombination events [16;32]. We constructed two phylogenetic trees using the standard 500 bp fragments of these six genes ("MLST tree") and the core genome. The structure of the "MLST tree" was in almost perfect agreement with the respective data of previous studies [22–24].

MLST is a good approach for rough classification of strains in phylogenetic groups, but by NGS the analysis of the core genome and the yield of higher resolution of phylogeny is possible. As the draft genomes of the entire ECOR collection are now available for the scientific community, extensive *in silico* approaches can be performed to investigate genomic regions of interest. Furthermore, the draft genomes were used to analyse the DNA acquisition combining *in silico* and *in vitro* experiments with a focus on three PAIs, namely the HPI, the *pks* and the *serU* island as well as the surrounding backbone genome. Due to the distribution of the three PAIs, we classified the analysed strains into distinct clusters named PAI-groups. We were able to demonstrate, that the isolates of the different PAI-groups carried distinct PAI subtypes regarding the respective islands. Also the neighbouring backbone regions were nearly identical within each PAI-group. In contrast, the housekeeping genes scattered around the genome showed no relationship among the strains within each group. This evidenced the same origin of these PAIs and the directly adjacent backbone genome underscoring their horizontal "*en bloc*" transfer.

Our 3-way comparison of phylogenetic trees using ML with bootstrap, ML with Bayesian inference and NJ showed differences in clustering ECOR strains, but the conclusions to the horizontal PAI transfer was supported by all three methods.

In order to reproduce the "*en bloc*" transfer indicated by our *in silico* data, we constructed two donors: one harbouring the HPI and the *pks* island (NU14 HPI-Cm F'; PAI-group 2a) and one harbouring all three PAIs (536 HPI-Cm F'; PAI-group 3). To apply a transfer of immobile PAIs, we used an F' plasmid which transfers the donors’ DNA to the recipients leading to integration. The resulting transconjugants were fully sequenced and the NGS data enabled us to investigate exactly the size of the integrated donor DNA. We could transfer up to 470.5 kb, which is almost 10% of the whole *E*. *coli* genome. These transfer events showed that in most cases all PAIs were transmitted together with the directly adjacent backbone. Interestingly, the *pks* island was only transferred into 91.4% of transconjugants from PAI-group 3. This indicated that the PAIs of the different PAI-groups were not always completely transferred. In contrast, we never observed partial transfer of the islands leading to fragmented PAIs. This was probably due to the required sequence homology of donor’s and recipient’s DNA, suggesting an "all or nothing" transfer.

Of note, the tetracycline-resistant transconjugants were able to further spread the received PAIs with higher conjugation efficiency. This could be the reason for the broad distribution of the *E*. *coli* HPI although this PAI is not self-transferable. Interestingly, the ICE-type of the HPI, which is still mobile, is less present in the *E*. *coli* species [5;33].

In the phylogenetic analyses of the *pks* island and the inter-PAI regions, the strain ECOR65 from PAI-group 2a did not cluster together with strains of the respective PAI-group, but could be assigned to PAI-group 3 strains. Instead, the HPI sequence resembled those of PAI-group 1, which were scattered over the phylogenetic tree of the HPI. One possible explanation would be that ECOR65 gained all three PAIs and the neighbouring backbone from a member of PAI-group 3 and subsequently lost the *serU* island in a deletion event. It was reported that the region surrounding the *serU* island and the HPI is a hotspot of recombination [34] and a "bastion of polymorphism" [34;35]. Nevertheless, this hypothesis doesn't explain the divergence between the ECOR65-HPI and the HPIs of PAI-group 3 strains. Another explanation is based on our findings of the *in vitro* approach. According to the phylogenetic tree of the HPI (Fig 3), ECOR65 was a former PAI-group 1 isolate. Then, a PAI-group 3 strain might have transferred only the *pks* island instead of three PAIs, representing an incomplete transfer event of the respective islands of the PAI group. This would lead to a clonal HPI subtype of PAI-group 1 and a clonal *pks* island subtype with surrounding backbone genome of PAI-group 3. This hypothesis is supported by the fact that especially the inter-PAI region between the HPI and the *pks* island were identical (Fig 8).

For the first time, we were able to reconstruct the HGT of large genomic regions by a combination of *in silico* and *in vitro* experiments due to NGS. The results showed that the exchange of immobile *E*. *coli* PAIs between *E*. *coli* isolates also influences the genomic backbone. Further data have to follow to completely understand the dimension of this transfer.

## Supporting information

S1 Fig Radial tree of the six housekeeping gene fragments. The radial tree of the six housekeeping gene fragments (*trpA*, *trpB*, *pabB*, *putP*, *icd* and *polB*) from the ECOR collection and strains S107, S108 and 536. The scale bar represents the number of SNPs per nucleotide. The node colour represents the distribution of the PAIs. The node shapes show the phylogenetic group according to the triplex PCR [2]. a) Tree performed by PhyML using the Maximum Likelihood algorithm with bootstrap. b) Tree performed by CLC Genomics Workbench using the Neighbour-Joining algorithm.
(TIF)

S2 Fig The phylogenetic tree of the entire HPI. All strains are at least HPI-positive. The text and dot colour represents the PAI-group and the dot shape the phylogenetic group. Except strain ECOR65 (asterisk) from PAI-group 2a, all members of PAI-groups 2a (blue), 2b (green) and 3 (red) showed a HPI subtype specific for their group. The scale bar represents the percentage of SNPs per nucleotide. a) The utilized algorithm was Maximum Likelihood with bootstrap performed by PhyML. b) The utilized algorithm was Neighbour-Joining performed by CLC Genomics Workbench.
(TIF)

S3 Fig The phylogenetic tree of the entire *serU* island. All strains are at least HPI- and *serU* island-positive. The text and dot colour represents the PAI-group and the dot shape the phylogenetic group. The members of PAI-groups 2b (green) and 3 (red) showed a *serU* island subtype specific for their group. The scale bar represents the percentage of SNPs per nucleotide. a) The algorithm which was used by PhyML was Maximum Likelihood with bootstrap. b) The algorithm which was used by CLC Genomics Workbench was Neighbour-Joining.
(TIF)

S4 Fig The phylogenetic tree of the entire *pks* island. All strains are at least HPI- and *pks* island-positive. The text and dot colour represents the PAI-group and the dot shape the phylogenetic group. Except strain ECOR65 (asterisk) from PAI-group 2a, all members of PAI-groups 2a (blue) and 3 (red) showed a *pks* island subtype specific for their group. The scale bar represents the number of SNPs per nucleotide. a) The algorithm we used was Maximum Likelihood with bootstrap performed by PhyML. b) The algorithm we used was Neighbour-Joining performed by CLC Genomics Workbench.
(TIF)

S5 Fig Phylogenetic tree of region A. The inter-PAI region between the *serU* island and the HPI (region A) is shown as phylogenetic tree. The text and dot colour represents the PAI-group and the dot shape the phylogenetic group. The scale bar represents the percentage of SNPs per nucleotide. a) The algorithm which was used by PhyML was Maximum Likelihood with bootstrap. b) The algorithm which was used by CLC Genomics Workbench was Neighbour-Joining.
(TIF)

S6 Fig Phylogenetic tree of region B. The dendrogram of the inter-PAI region between the HPI and the *pks* island (region B). The text and dot colour represents the PAI-group and the dot shape the phylogenetic group. The scale bar represents the number of SNPs per nucleotide. a) The algorithm which was used by PhyML was Maximum Likelihood with bootstrap. b) The algorithm which was used by CLC Genomics Workbench was Neighbour-Joining.
(TIF)

S1 Table NGS accessions. (XLSX)

S2 Table NGS parameters. (DOCX)

S3 Table ECOR phylogeny. (XLSX)
