# Supplementary figures and images for: Investigation of horizontal gene transfer of pathogenicity islands in Escherichia coli using next-generation sequencing
Source: PLoS One. 2017 Jul 21;12(7):e0179880. doi: 10.1371/journal.pone.0179880 (PMC5521745; doi:10.1371/journal.pone.0179880)

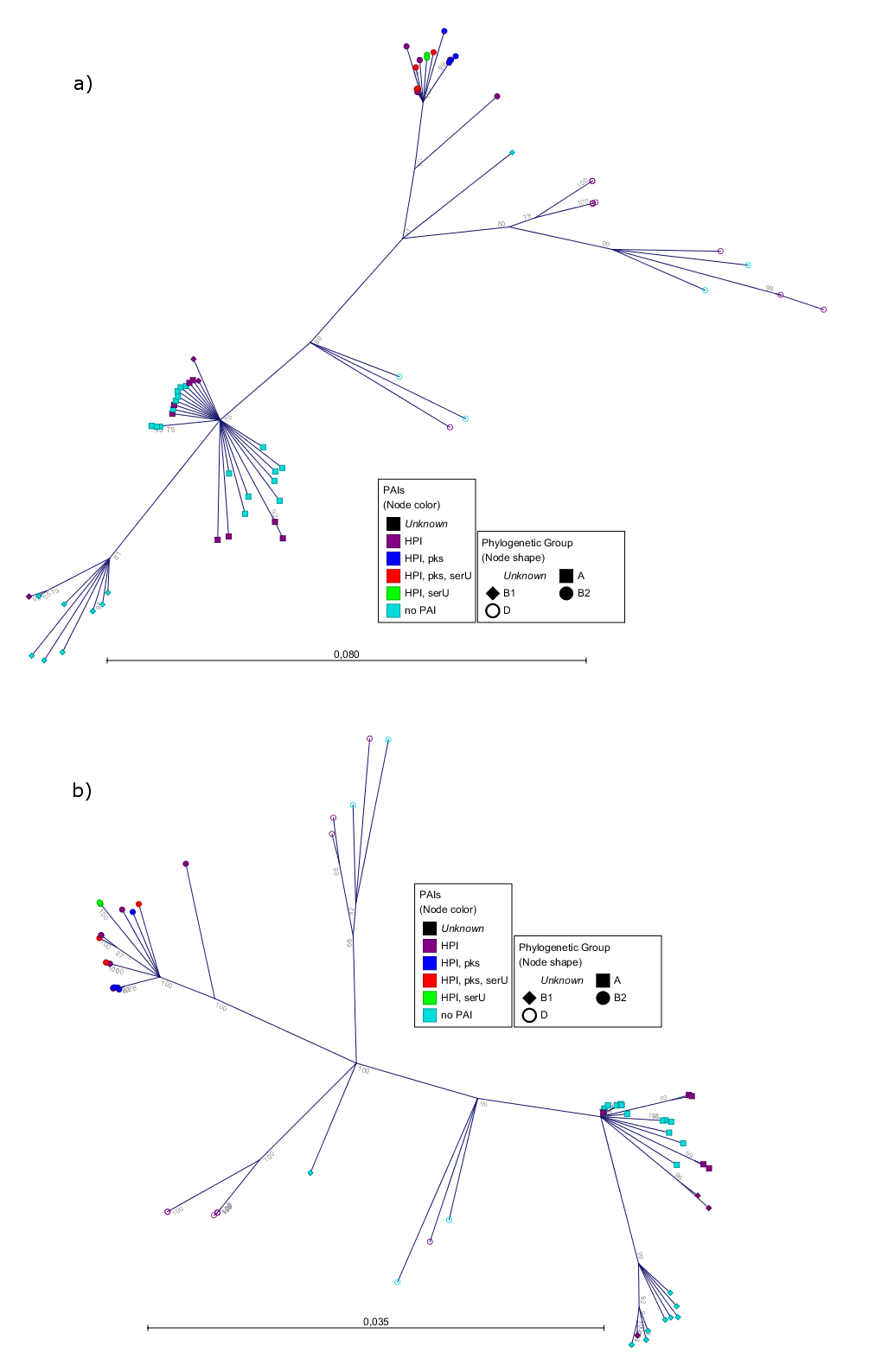

Supplement: S1 Fig — The radial tree of the six housekeeping gene fragments (trpA, trpB, pabB, putP, icd and polB) from the ECOR collection and strains S107, S108 and 536. The scale bar represents the number of SNPs per nucleotide. The node colour represents the distribution of the PAIs. The node shapes show the phylogenetic group according to the triplex PCR [2]. a) Tree performed by PhyML using the Maximum Likelihood algorithm with bootstrap. b) Tree performed by CLC Genomics Workbench using the Neighbour-Joining algorithm. (TIF) [file pone.0179880.s001.tif]

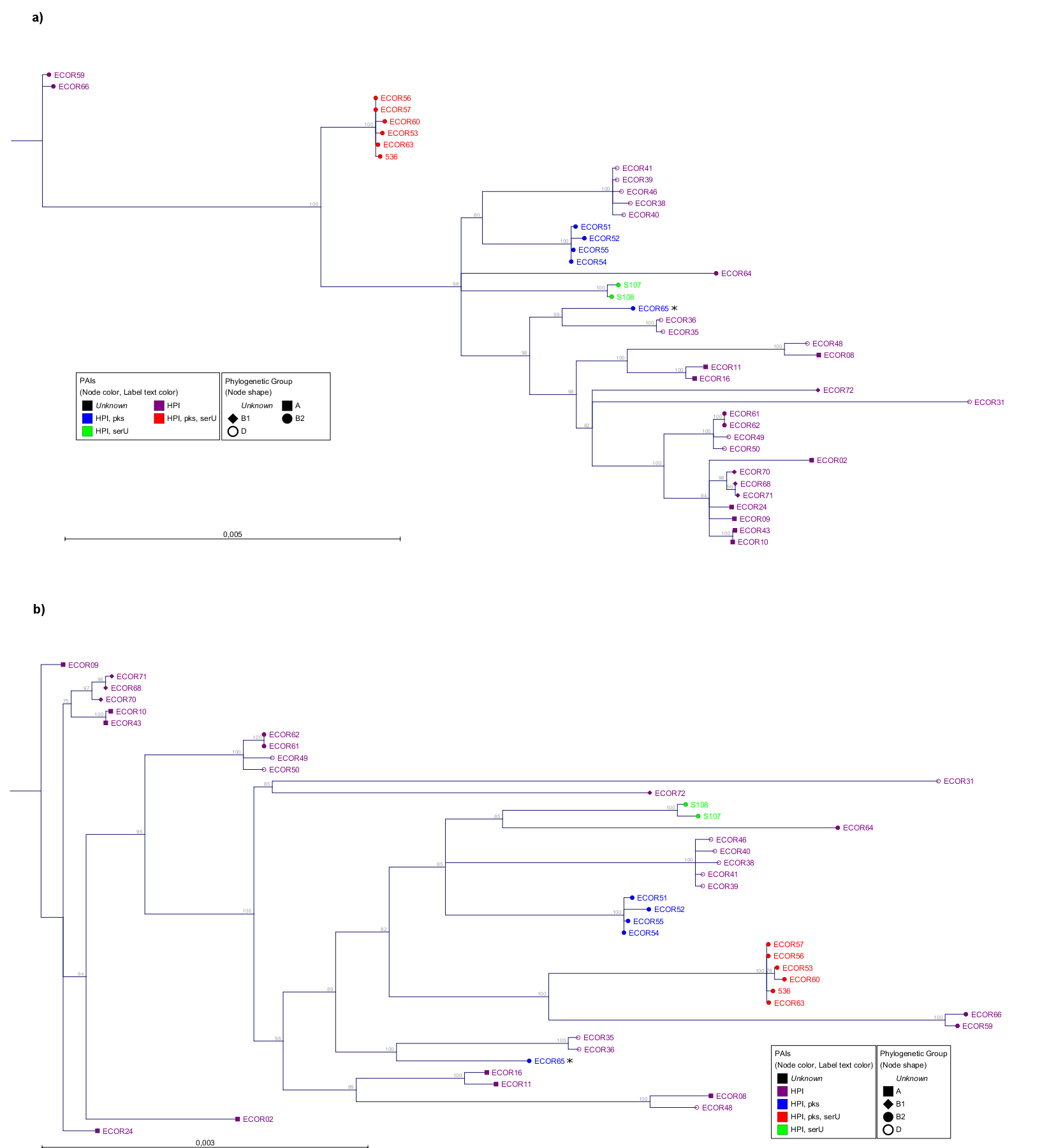

Supplement: S2 Fig — All strains are at least HPI-positive. The text and dot colour represents the PAI-group and the dot shape the phylogenetic group. Except strain ECOR65 (asterisk) from PAI-group 2a, all members of PAI-groups 2a (blue), 2b (green) and 3 (red) showed a HPI subtype specific for their group. The scale bar represents the percentage of SNPs per nucleotide. a) The utilized algorithm was Maximum Likelihood with bootstrap performed by PhyML. b) The utilized algorithm was Neighbour-Joining performed by CLC Genomics Workbench. (TIF) [file pone.0179880.s002.tif]

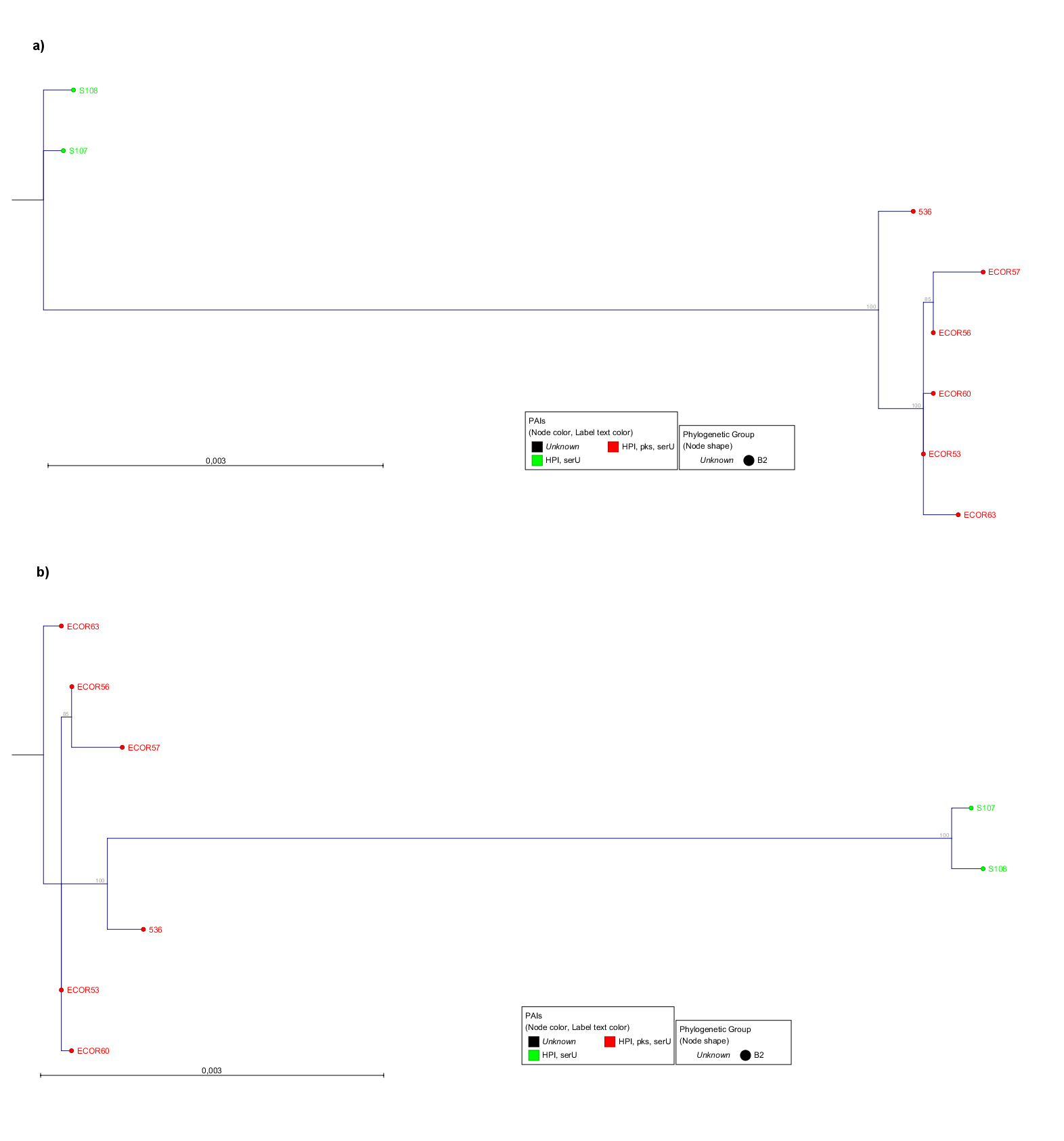

Supplement: S3 Fig — All strains are at least HPI- and serU island-positive. The text and dot colour represents the PAI-group and the dot shape the phylogenetic group. The members of PAI-groups 2b (green) and 3 (red) showed a serU island subtype specific for their group. The scale bar represents the percentage of SNPs per nucleotide. a) The algorithm which was used by PhyML was Maximum Likelihood with bootstrap. b) The algorithm which was used by CLC Genomics Workbench was Neighbour-Joining. (TIF) [file pone.0179880.s003.tif]

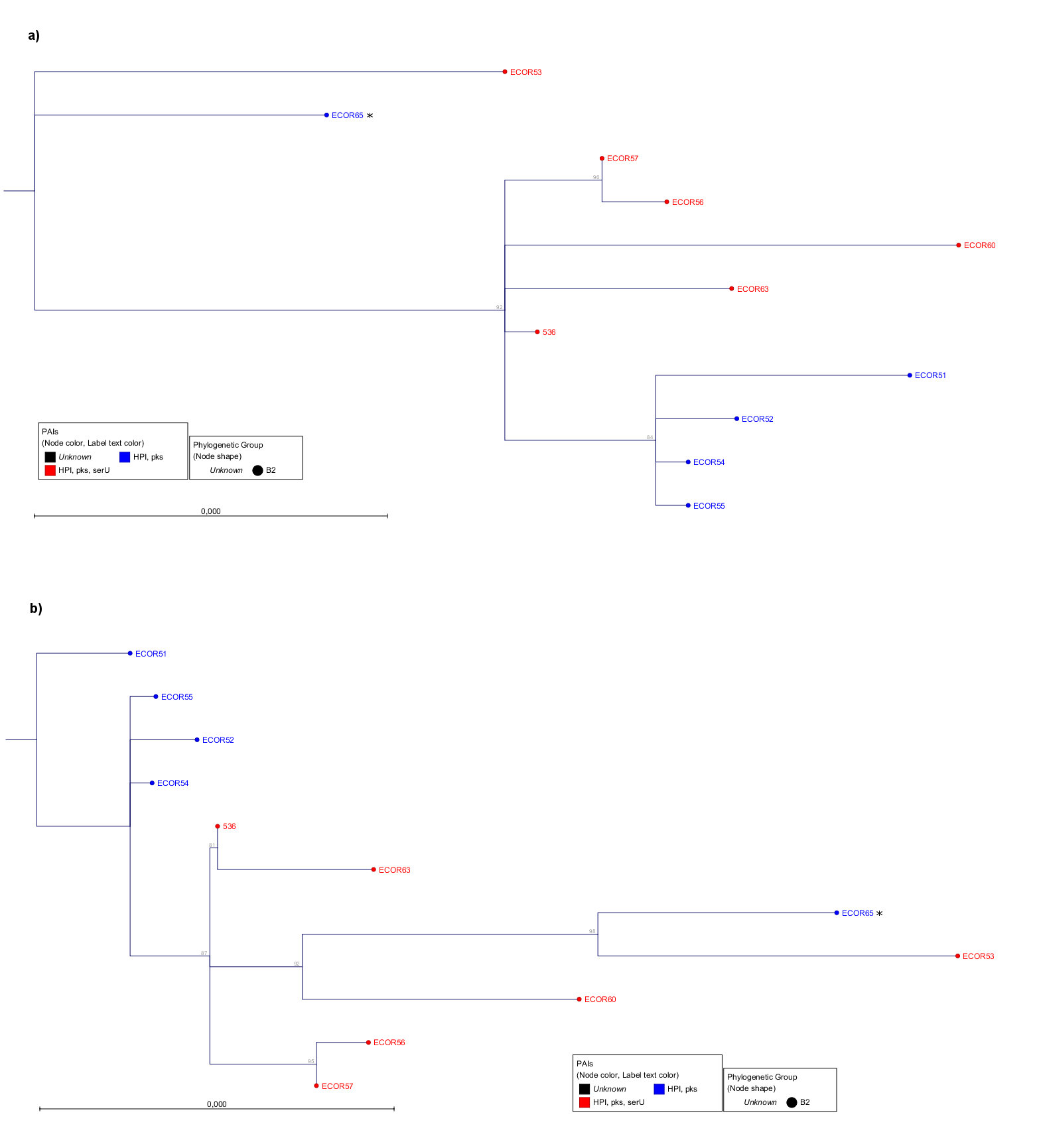

Supplement: S4 Fig — All strains are at least HPI- and pks island-positive. The text and dot colour represents the PAI-group and the dot shape the phylogenetic group. Except strain ECOR65 (asterisk) from PAI-group 2a, all members of PAI-groups 2a (blue) and 3 (red) showed a pks island subtype specific for their group. The scale bar represents the number of SNPs per nucleotide. a) The algorithm we used was Maximum Likelihood with bootstrap performed by PhyML. b) The algorithm we used was Neighbour-Joining performed by CLC Genomics Workbench. (TIF) [file pone.0179880.s004.tif]

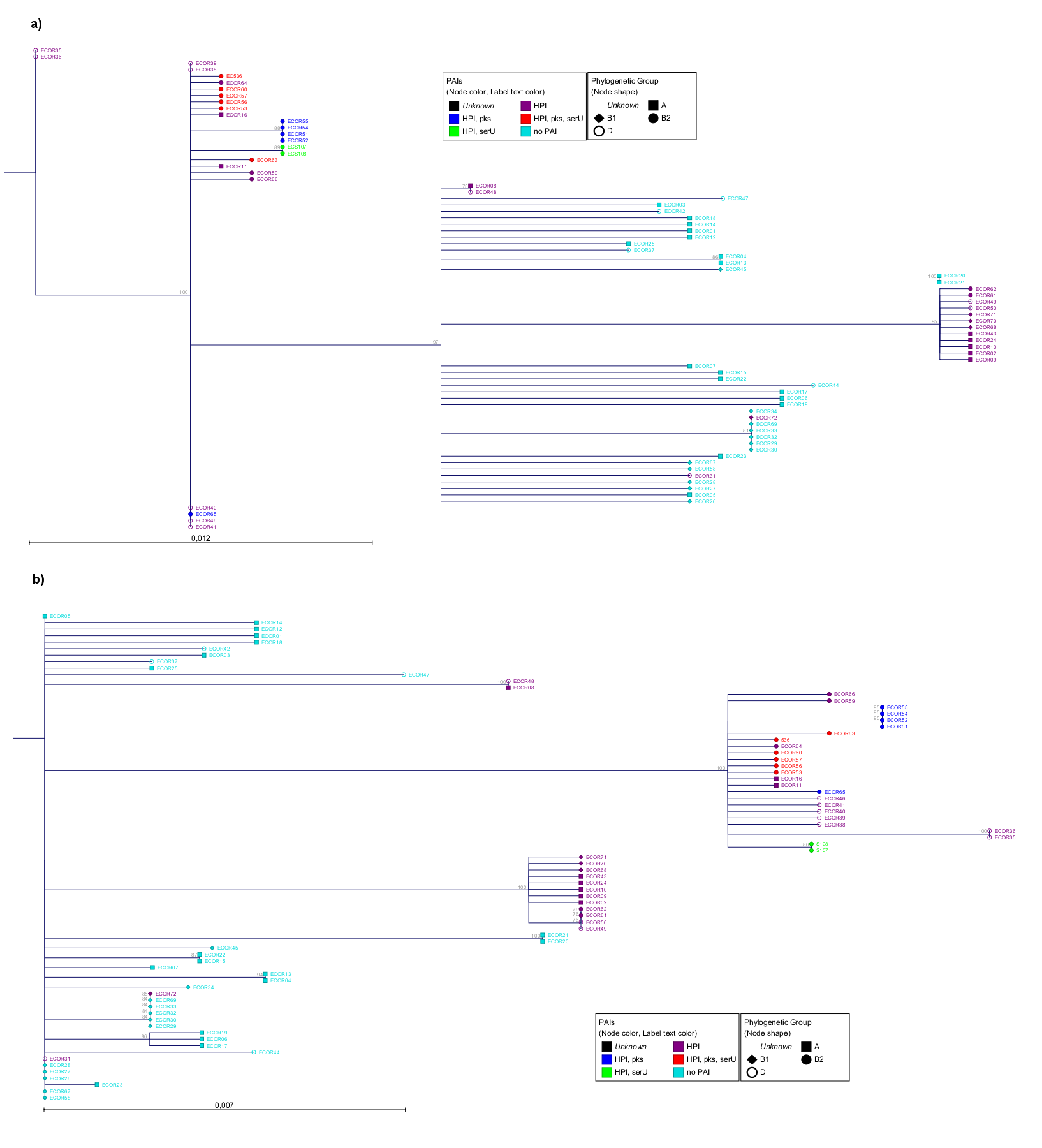

Supplement: S5 Fig — The inter-PAI region between the serU island and the HPI (region A) is shown as phylogenetic tree. The text and dot colour represents the PAI-group and the dot shape the phylogenetic group. The scale bar represents the percentage of SNPs per nucleotide. a) The algorithm which was used by PhyML was Maximum Likelihood with bootstrap. b) The algorithm which was used by CLC Genomics Workbench was Neighbour-Joining. (TIF) [file pone.0179880.s005.tif]

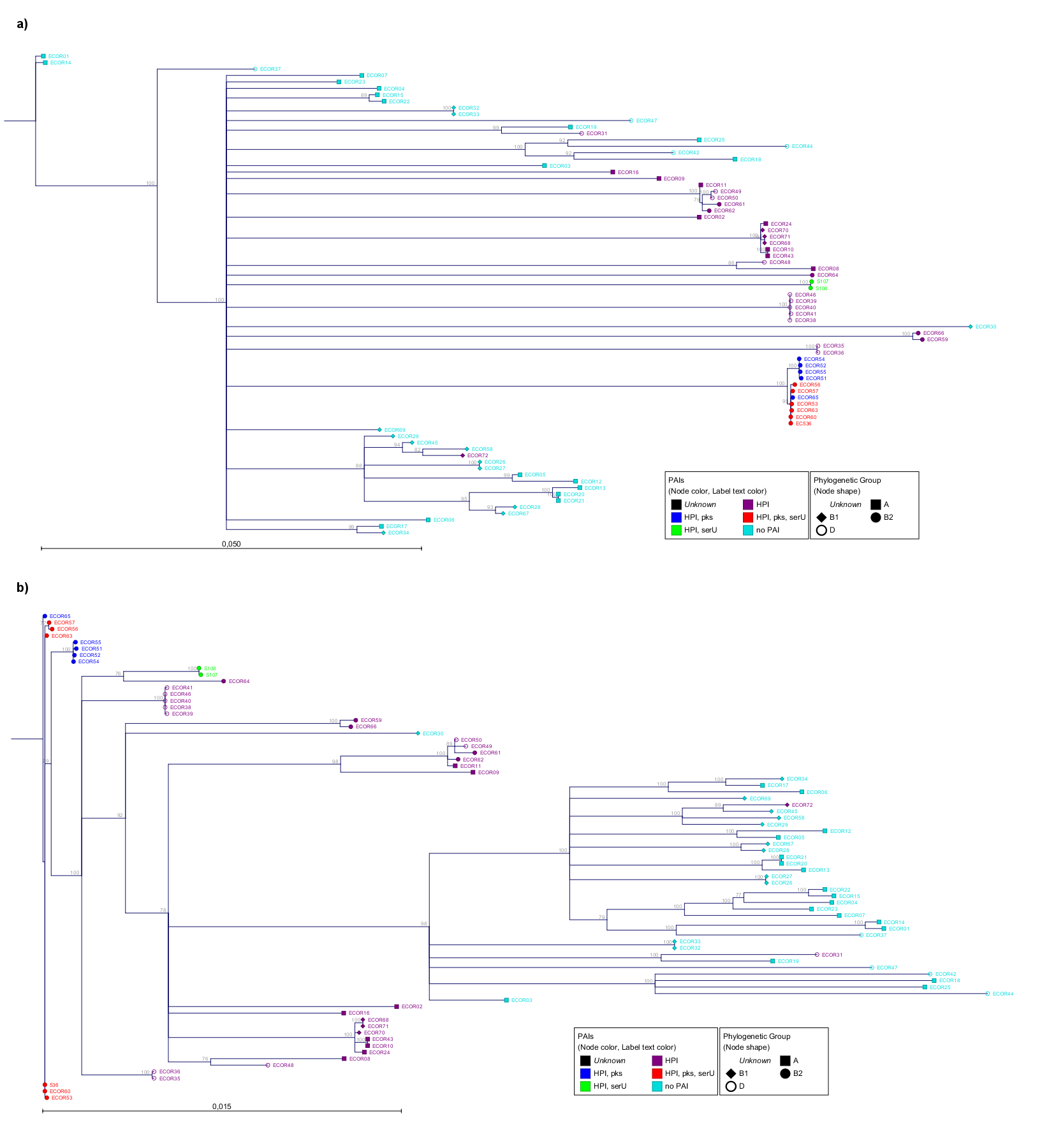

Supplement: S6 Fig — The dendrogram of the inter-PAI region between the HPI and the pks island (region B). The text and dot colour represents the PAI-group and the dot shape the phylogenetic group. The scale bar represents the number of SNPs per nucleotide. a) The algorithm which was used by PhyML was Maximum Likelihood with bootstrap. b) The algorithm which was used by CLC Genomics Workbench was Neighbour-Joining. (TIF) [file pone.0179880.s006.tif]
