# Supplementary material for: Investigation of horizontal gene transfer of pathogenicity islands in Escherichia coli using next-generation sequencing
Source: PLoS One. 2017 Jul 21;12(7):e0179880. doi: 10.1371/journal.pone.0179880 (PMC5521745; doi:10.1371/journal.pone.0179880)
Supplement: S2 Table — (DOCX) [file pone.0179880.s008.docx]

The raw reads were imported as Illumina data (parameters: "Remove failed reads", "Quality scores: NCBI/Sanger or Illumina Pipeline 1.8 and later") to CLC Genomics Workbench 6.5 (CLC bio; Aarhus, Denmark).

To trim the sequences the parameters "Ambiguous trim", "Ambiguous limit: 2", "Quality trim", "Quality limit: 0.05", "Maximum number of nucleotides in reads: 1000" and "Minimum number of nucleotides in reads: 15" were performed.

For the *de novo* assemblies the parameters "Mapping mode: Map reads back to contigs", "Update contigs", "Automatic bubble size", "Bubble size: 50", "Minimum contig length: 200", "Automatic word size: 20", "Perform scaffolding", "Auto-detect paired distances", "Mismatch cost: 2", "Insertion cost: 3", "Deletion cost: 3", "Length fraction: 0.5", "Similarity fraction: 0.8)", "Alignment mode: local", "Colorspace alignment" and "Colorspace error cost: 3" were performed.

The alignments were performed using the CLC Genomics Workbench 6.5 (parameters: "Gap open cost: 10", "Gap extension cost: 1" and "Less accurate").

The phylogenetic trees (Maximum Likelihood (ML) with bootstraps and with Bayesian branch support) were constructed using the online tool PhyML 3.0.

The parameters for the ML tree with bootstrap were “JC69”, “Proportion of invariable sites: 0.0”, “Number of substitution rate categories: 4”, “Type of tree improvement: NNI” and “Perform bootstrap: 100”).

The parameters for the ML tree with Bayesian branch support were “JC69”, “Proportion of invariable sites: 0.0”, “Number of substitution rate categories: 4”, “Type of tree improvement: NNI” and “Fast likelihood-based method: aBayes”).

The CLC software was also used for the in silico MLST to create phylogenetic trees applying the Neighbour Joining algorithm (parameters: "Neighbour Joining", "Jukes-Cantor", "Perform bootstrap analysis" and "Replicates: 100").
